# Supplementary material for: AI-based search for convergently expanding, advantageous mutations in SARS-CoV-2 by focusing on oligonucleotide frequencies
Source: PLoS One. 2022 Aug 31;17(8):e0273860. doi: 10.1371/journal.pone.0273860 (PMC9432735; doi:10.1371/journal.pone.0273860)
Supplement: S1 Fig — All 1169 heatmaps are presented along with 20-mer sequences. (PDF) [file pone.0273860.s001.pdf]

S1 Fig. Heatmap patterns. All 1169 heatmaps are presented along with 20-mer sequences.

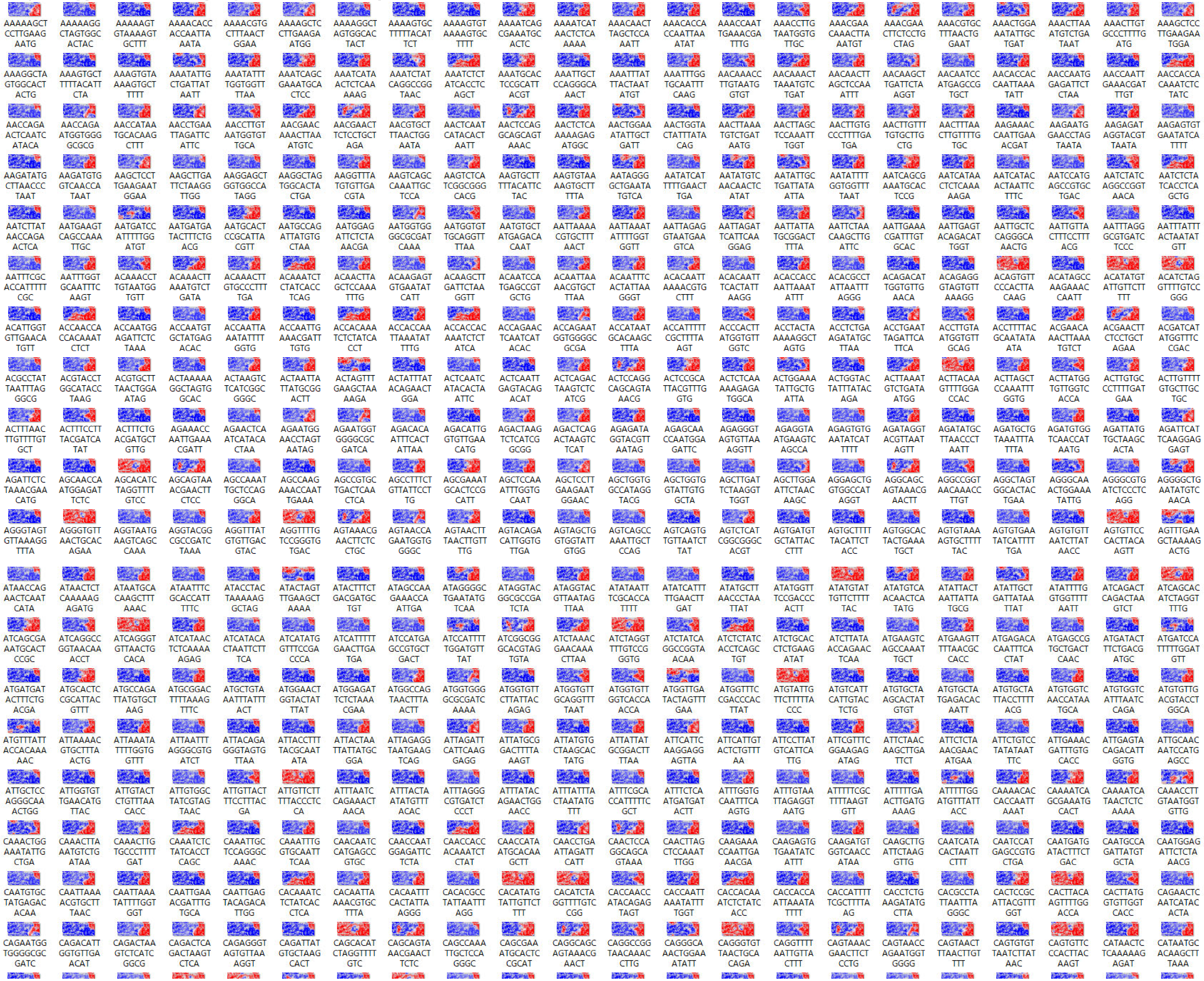

CATAGCCA AGAAACC AATTG	CATAGGTA CGGCGCCG ATCT	CATATGGT TTCCGACC CACT	CATATGTA TTGTTCTTT TTA	CATCAGCA CATCTAGG TTTT	CATCGGCG GGCACGTA GTGT	CATCTAAA CGAACAA ACTTA	CATCTAGG TTTTGTCCG GGT	CATGAGCC GTGCTGAC TCAA	CATGTGGT CATTTAAT CCAG	CATTCATT GTACTCTG TTTA	CATTCGTTT CGGAAGA GATA	CATTGGTG TTGAACAT GTTA	CATTGTAC TCTGTTTAA CAC	CATTTAAT CCAGAAAC TAAC	CATTTGTA ATTAGAGG TAAT	CATTTTCG CTTTAAGT GT	CATTTTGA ACTTGATG AAA	CATTTTGG ATGTTTATT AC	CCAAAATC ATAACTCT CAAA	CCAAATTG CTCCAGGG CAAA	CCAAATTT GGTGCAAT TTCA
CCAACCAC CACAAATC TCTA	CCAAGAA ACCAATTG AAACG	CCAATGGA GATTCTCT AAAC	CCAATGTG CTATGAGA CACA	CCAATTAA ATATTTTG GTGG	CCAATTGA AACGATTT GTGC	CCACAAAT CTCTATCA CCTC	CCACCAAT TAAATATT TTGG	CCACCACA AATCTCTA TCAC	CCACTTAC AAGTTTTG GACC	CCACTTAT GGTGTTGG TCAC	CCAGAACT CAATCATA CACT	CCAGAATG GTGGGGCG CGAT	CCAGATTA TGTGCTAA GCAC	CCAGGCAG CAGTAAAC GAAC	CCAGGGCA AACTGGAA ATAT	CCAGTAAC TTAACTTG TTT	CCATAATG CACAAGCT TTAA	CCATAGGT ACGGCGCC GATC	CCATGAGC CGTGCTGA CTCA	CCATGTGG TCATTTAAT CCA	CCATTTTTC GCTTTTAA GTG
CCATTTTG GATGTTTA TTA	CCCACTTA CAAGTTTT GGAC	CCCACTTA TGGTGTTG GTCA	CCCTTTTGA TGAAGTTT TTA	CCGACCCA CTTATGGT GTTG	CCGGTAAC AAACCTTG TAAT	CCGTGCTG ACTCAACT CAGG	CCTACTAA AAAGGCTA GTGG	CCTATATA ATTTCGCA CCAT	CCTATTAA TTTAGGGC GTGA	CCTCTGAA GATATGCT TAAC	CCTGAATT AGATTCAT TCAA	CCTTATGTC ATTCATTGT AC	CCTTGAAG AATGGAAC CTAG	CCTTGTAA TGGTGTTG CAGG	CCTTTACG ATCATATG GTTT	CCTTTCTGT TATTCCTTG TT	CCTTTTACG CAATATAA TAG	CCTTTTGAT GAAGTTTT TAA	CGAAATGC ACTCCGCA TTAC	CGAACAA ACTTAAAT GTCTG	CGAACTTC TCCTGCTA GAAT
CGACCCAC TTATGGTG TTGG	CGATCATA TGGTTTCC GACC	CGCACCAT TTTTCGCTT TTA	CGCCTATT AATTTAGG GCGT	CGCGTTCC ATGTGGTC ATTT	CGCTTCCA AAATCATA ACTC	CGCTTTTA AGTGTTAT GGAG	CGGAAGA GATAGGTA CGTTA	CGGACTTT TAAAGTTT CCAT	CGGTAACA AACCTTGT AATG	CGTACCTG GCATACCT AAGG	CGTGCTGA CTCAACTC AGGC	CGTTCCAT GTGGTCAT TTAA	CGTTTCGG AAGAGAT AGGTA	CTAAAAAG GCTAGTGG CACT	CTAAACGA ACAAACTT AAAT	CTAAACGA ACATGAAA ATTA	CTAACAAG CTTGATTCT AAG	CTAAGTCT CATCGGCG GGCA	CTAATTATT ATGCGGAC TTT	CTACAGTG TTCCCACTT ACA	CTACTAAA AAGGCTAG TGGC
CTAGGTTTT GTCCGGGT GTG	CTAGTGGC ACTACTGA AATG	CTAGTTTG AAGCTAAA AGAC	CTATATAA TTTCGCAC CATT	CTATCAGG CCGGTAAC AAAC	CTATGAGA CACAATTT CACT	CTATTAATT TAGGGCGT GAT	CTATTACCT TTTACGCA ATA	CTATTCCTT ATGTCATT CAT	CTATTTATA CAGAACTG GAA	CTCAATCA TACACTAA TTCT	CTCAATGA TGATACTTT CTG	CTCAATTG AGTACAGA CATT	CTCAGACT AAGTCTCA TCGG	CTCATCGG CGGGCACG TAGT	CTCCAAAT TTGGTGCA ATTT	CTCCAGGC AGCAGTAA ACGA	CTCCAGGG CAAACTGG AAAT	CTCCGCAT TACGTTTG GTGG	CTCCTTGA AGAATGG AACCT	CTCTAAAC GAACATGA AAAT	CTCTACAG TGTTCCCA CTTA
CTCTCAAA AAGAGAT GGCAA	CTCTGAAG ATATGCTT AACC	CTGAAGAT ATGCTTAA CCCT	CTGAATAT GTCAACAA CTCA	CTGAATTA GATTCATT CAAG	CTGCACCT CTGAAGAT ATGC	CTGGAAAT ATTGCTGA TTAT	CTGGCAGT AACCAGA ATGGT	CTGGTACT ATTTATAC AGAA	CTGGTGGC CATAGGTA CGGC	CTGGTGGT ATTGTGGC TATC	CTGTAAAT TATTTACT AAT	CTGTCCTAT ATAATTTC GCA	CTGTTATTC CTTGTTTA AT	CTGTTCTTT ATCAGGGT GTT	CTGTTTAAT AGGGGCTG AAT	CTTAAAAA GCTCCTTG AAGA	CTTAAATG TCTGATAA TGGA	CTTACAAG TTTTGGAC CACT	CTTAGCTC CAAATTTG GTGC	CTTATAAC CAGAACTC AATC	CTTATGGT GTTGGTCA CCAA
CTTATGTC ATTCATTGT ACT	CTTATTAC AGAGGGT AGTGT	CTTCCAAA ATCATAAC TCTC	CTTGAAGA ATGGAACC TAGT	CTTGGAAT TCTAACAA GCTT	CTTGTAAT GGTGTTGC AGGT	CTTGTGCC CTTTGATG AAG	CTTTAACTT GTTTGTGC TT	CTTTACCA ACCACCAC AAAT	CTTTACGA TCATATGG TTTC	CTTTATCA GGGTGTTA ACTG	CTTTCCTTT ACGATCAT ATG	CTTTCTGAC GATGCTGT TGT	CTTTCTGTT ATTCCTTGT TT	CTTTGCAA CCTGAATT AGAT	CTTTTACGC AATATAAT AGA	CTTTTGATG AAGTTTTT AAC	CTTTTTACA TTCTACCAT CT	CTTTTTACC CTCCAGAT GAG	CTTTTTAGC CTTTCTGTT AT	GAAACCA ATTGAAAC GATTT	GAAATATT GCTGATTA TAAT
GAAATCTA TCAGGCCG GTAA	GAAATGCA CTCCGCAT TACG	GAACAAA CTTAAATG TCTGA	GAACTCAA TCATACAC TAAT	GAACTGGT ACTATTTAT ACA	GAAGAAT GGAACCTA GTAAT	GAAGAGA TAGGTACG TTAAT	GAAGATAT GCTTAACC CTAA	GAAGCTAA AAGACTGT GTTA	GAAGGTTT ATGTGTTG ACGT	GAAGTCAG CCAAATTG CTCC	GAATATCA TTTTGAAC TTG	GAATATGT CAACAACT CATA	GAATGGA ACCTAGTA ATAGG	GAATGGTG GGGCGCG ATCAA	GAATTAGA TTCATTCA AGGA	GAATTCTA ACAAGCTT GATT	GACACAAT TTCACTATT AAG	GACATAGC CAAGAAA CCAAT	GACATTGG TGTTGAAC ATGT	GACCCACT TATGGTGT TGGT	GACGTACC TGGCATAC CTAA
GACTAAGT CTCATCGG CGGG	GACTCAGA CTAAGTCT CATC	GAGACAC AATTTCAC TATTA	GAGATAG GTACGTTA ATAGT	GAGATTCT CTAAACGA ACAT	GAGCAACC AATGGAG ATTCT	GAGCCGTG CTGACTCA ACTC	GAGCTGGT GGCCATAG GTAC	GAGGGTA GTGTTAAA GGTTT	GAGGTAAT GAAGTCAG CCAA	GAGTACAG ACATTGGT GTTG	GAGTGTGA ATATCATTT TTG	GATACTAG TTTGAAGC TAAA	GATACTTT CTGACGAT GCTG	GATAGGTA CGTTAATA GTTA	GATATGCT TAACCCTA ATTA	GATATTAC TAATTATT ATGC	GATCATAT GGTTTCCG ACCC	GATCCATT TTTGGATG TTTA	GATCTGCA CCTCTGAA GATA	GATGAAGT TTTTAACG CCAC	GATGATAC TTTCTGAC GATG
GATGCTGT AAATTTAT TTAC	GATGGAAC TGGTACTA TTTA	GATGTGCT ATTACCTTT TAC	GATGTGGT CAACCATA ATGC	GATGTTTA TTACCACA AAAA	GATTATGT GCTAAGCA CTAT	GATTCATT CAAGGAG GAGTT	GATTCTCT AAACGAA CATGA	GCAAACTG GAAATATT GCTG	GCAACAAT CCATGAGC CGTG	GCAACCAA TGGAGATT CTCT	GCAACCTG AATTAGAT TCAT	GCACATCT AGGTTTTG TCCG	GCACCATT TTTCGCTTT TAA	GCACCTCT GAAGATAT GCTT	GCACTCCG CATTACGT TTGG	GCAGCAGT AAACGAA CTTCT	GCAGGTTT TAATTGTT ACTT	GCAGTAAA CGAACTTC TCCT	GCAGTAAC CAGAATGG TGGG	GCCAAATT GCTCCAGG GCAA	GCCAAGA AACCAATT GAAAC
GCCAGATT ATGTGCTA AGCA	GCCAGTAA CTTTAACTT GTT	GCCATAGG TACGGCGC CGAT	GCCCTTTTG ATGAAGTT TTT	GCCGGTAA CAAACCTT GTAA	GCCGTGCT GACTCAAC TCAG	GCCTATTA ATTTAGGG CGTG	GCCTTTGTG TTATTCCTT GT	GCGAAATG CACTCCGC ATTA	GCGCTTCC AAAATCAT AACT	GCGGACTT TTAAAGTT TCCA	GCGTTCCA TGTGGTCA TTTA	GCTAGTGG CACTACTG AAAT	GCTATGAG ACACAATT TCAC	GCTATTAC CTTTTACGC AAT	GCTCCAAA TTTGGTGC AATT	GCTCCTTG AAGAATG GAACC	GCTGAATA TGTCAACA ACTC	GCTGGTGG CCATAGGT ACGG	GCTGGTGG TATTGTGG CTAT	GCTGTAAA TTTATTTAC TAA	GCTGTTCTT TATCAGGG TGT
GCTTAAAA AGCTCCTT GAAG	GCTTCCAA AATCATAA CTCT	GCTTGATT CTAAGGTT GGTG	GCTTGGAA TTCTAACA AGCT	GCTTTTAA GTGTTATG GAGT	GCTTTTTAC ATTCTACC ATC	GCTTTTTAG CCTTTCTGT TA	GGAAATAT TGCTGATT ATAA	GGAACTGG TACTATTTA TAC	GGAAGAG ATAGGTAC GTTAA	GGAATTCT AACAAGCT TGAT	GGAGATTC TCTAAACG AACA	GGAGCTGG TGGCCATA GGTA	GGATGTTT ATTACCAC AAAA	GGCAAACT GGAAATAT TGCT	GGCAGCA GTAAACGA ACTTC	GGCAGTAA CCAGAATG GTGG	GGCCAGTA ACTTTAAC TTGT	GGCCATAG GTACGGCG CCGA	GGCCGGTA ACAAACCT TGTA	GGCGTGAT CTCCCTCA GGGT	GGCTAGTG GCACTACT GAAA
GGCTATCG TAGTAACA TGCC	GGCTGAAT ATGTCAAC AACT	GGGCAAA CTGGAAAT ATTGC	GGGCGTGA TCTCCCTCA GGG	GGGCTGAA TATGTCAA CAAC	GGGGCTGA ATATGTCA ACAA	GGGTAGTG TTAAAGGT TTAC	GGGTGTTA ACTGCACA GAAG	GGTAACAA ACCTTGTA ATGG	GGTAATGA AGTCAGCC AAAT	GGTACGGC GCCGATCT AAAG	GGTACTAT TTATACAG AACT	GGTATTGT GGCTATCG TAGT	GGTCAACC ATAATGCA CAAG	GGTCACCA ACCATACA GAGT	GGTCATTT AATCCAGA AACT	GGTGGCCA TAGGTACG GCGC	GGTGGGGC GCGATCAA AACA	GGTGGTAT TGTGGCTA TCGT	GGTGTTAA CTGCACAG AAGT	GGTGTTCTT ATTACAGA GGG	GGTGTTGC AGGTTTTA ATTG
GGTGTTGG TCACCAAC CATA	GGTTGATA CTAGTTTG AAGC	GGTTTATG TGTTGACG TACC	GGTTTCCG ACCCACTT ATGG	GGTTTTGTC CGGGTGTG ACC	GTAAAAGT GCTTTTTAC ATT	GTAAACGA ACTTCTCT GCT	GTAAATTT ATTTACTA ATAT	GTAACAAA CCTTGTAA TGGT	GTAACCAG AATGGTGG GGCG	GTAACTTT AACTTGTTT TGT	GTAATGAA GTCAGCCA AATT	GTAATGAT CCATTTTTG GAT	GTAATGGT GTTGCAGG TTTT	GTAATTAG AGGTAATG AAGT	GTACACAA TTAAAACG TGCT	GTACAGAC ATTGGTGT TGAA	GTACCTGG CATACCTA AGGA	GTACGGCG CCGATCTA AAGT	GTACTATTT ATACAGAA CTG	GTAGATGC TGTAAATT TATT	GTAGCTGG TGGTATTG TGGC
GTATTGTG GCTATCGT AGTA	GTATTGTTC TTTTTACCC TC	GTCAACCA TAATGCAC AAGC	GTCAATGC CAGATTAT GTGC	GTCACCAA CCATACAG AGTA	GTCAGCCA AATTGCTC CAGG	GTCAGTGT GTTAATCTT ATA	GTCATTCA TTGTACTCT GTT	GTCATTTA ATCCAGAA ACTA	GTCCTATA TAATTTCG CACC	GTCTCATC GGCGGGC ACGTA	GTGAATAT CATTTTTGA ACT	GTGATGGA ACTGGTAC TATT	GTGATGTG CTATTACCT TTT	GTGCCCTTT TGATGAAG TTT	GTGCTATG AGACACA ATTTC	GTGCTATT ACCTTTTAC GCA	GTGCTTTTT ACATTCTA CCA	GTGCTTTTT AGCCTTTCT GT	GTGGCCAT AGGTACGG CGCC	GTGGCTAT CGTAGTAA CATG	GTGGTATT GTGGCTAT CGTA
GTGGTCAA CCATAATG CACA	GTGGTCAT TTAATCCA GAAA	GTGTAAAA GTGCTTTTT ACA	GTGTGAAT ATCATTTTT GAA	GTGTGTTA ATCTTATA ACCA	GTGTTAAC TGCACAGA AGTC	GTGTTAAT CTTATAAC CAGA	GTGTTCCC ACTTACAA GTTT	GTGTTCTTA TTACAGAG GGT	GTGTTGAC GTACCTGG CATA	GTGTTGCA GGTTTTAA TTGT	GTGTTGGT CACCAACC ATAC	GTTAATCTT ATAACCAG AAC	GTTACTTTC CTTTACGA TCA	GTTATCAG ACTCAGAC TAAG	GTTATGGC CAGTAACT TTAA	GTTATTCCT TGTTTTAAT TA	GTTCCATG TGGTCATTT AAT	GTTCCCAC TTACAAGT TTTG	GTTCTTATT ACAGAGG GTAG	GTTCTTTAT CAGGGTGT TAA	GTTCTTTTT ACCCTCCA GAT
GTTGACGT ACCTGGCA TACC	GTTGATAC TAGTTTGA AGCT	GTTGCAGG TTTTAATTG TTA	GTTGCTGTT CTTTATCA GGG	GTTGGTCA CCAACCAT ACAG	GTTGTCAA TGCCAGAT TATG	GTTTAATA GGGGCTGA ATAT	GTTTATGT GCTAAGCA ACCT	GTTTCCGA CCCACTTA TGGT	GTTTCGGA AGAGATA GGTAC	GTTTGAAG CTAAAAGA CTGT	GTTTTGTCC GGGTGTGA CCG	GTTTTTGTA CACAATTA AAA	TAAAAAGC TCCTTGAA GAAT	TAAAAAG GCTAGTGG CACTA	TAAAAAGT GTAAAAGT GCTT	TAAAACGT GCTTTAAC TGGA	TAAAAGTG CTTTTTACA TTC	TAAACAAC TTAGCTCC AAAT	TAAACGAA CAAACTTA AATG	TAAACGAA CTTCTCCTG CTA	TAAATATT TTGGTGGT TTTA
TAAATGTC TGATAATG GACC	TAAATTTA TTTACTAAT ATG	TAACAAAC CTTGTAAT GGTG	TAACAAGC TTGATTCTA AGG	TAACCAGA ACTCAATC ATAC	TAACCAGA ATGGTGGG GCGC	TAACTCTC AAAAAGA GATGG	TAACTTGTT TTGTGCTTG CT	TAACTTTA ACTTGTTTT GTG	TAAGTCTC ATCGGCGG GCAC	TAATAGGG GCTGAATA TGTC	TAATCCAG AAACTAAC ATTC	TAATCTTAT AACCAGA ACTC	TAATGAAG TCAGCCAA ATTG	TAATGATC CATTTTTGG ATG	TAATGCAC AAGCTTTA AACA	TAATGGTG TTGCAGGT TTTA	TAATTAGA GGTAATGA AGTC	TAATTATT ATGCGGAC TTTT	TAATTTAG GGCGTGAT CTCC	TAATTTCG CACCATTTT TCG	TACAAGAG TGTGAATA TCAT
TACACAAT TAAAACGT GCTT	TACAGACA TTGGTGTT GAAC	TACAGAGG GTAGTGTT AAAG	TACAGTGT TCCCACTT ACAA	TACCAACC ACCACAAA TCTC	TACCAATG TGCTATGA GACA	TACCTACT AAAAAGG CTAGT	TACCTTTTA CGCAATAT AAT	TACGATCA TATGGTTTC CGA	TACTAAAA AGGCTAGT GGCA	TACTAATT ATTATGCG GACT	TACTAGTTT GAAGCTAA AAG	TACTATTTA TACAGAAC TGG	TACTTTCCT TTACGATC ATA	TACTTTCTG ACGATGCT GTT	TAGAGGTA ATGAAGTC AGCC	TAGATGCT GTAAATTT ATTT	TAGATTCA TTCAAGGA GGAG	TAGCCAAG AAACCAAT TGAA	TAGCCTTTC TGTTATTCC TT	TAGCTCCA AATTTGGT GCAA	TAGCTGGT GGTATTGT GGCT
TAGCTTGG	TAGGGCGT	TAGGGGCT	TAGGTACG	TAGGTACG	TAGGTTTT	TAGTAGCT	TAGTGATG	TAGTGGCA	TAGTTTGA	TATAACCA	TATAATTC	TATACCTA	TATATAAT	TATCAGAC	TATCAGGC	TATCAGGG	TATCATTTT	TATGAGAC	TATGCGGA	TATGCTTA	TATGGCCA

[illegible]<br>AATTCTAA<br>CAAG	[illegible]<br>GATCTCCC<br>TCAG	[illegible]<br>GAATATGT<br>CAAC	[illegible]<br>GCGCCGAT<br>CTAA	[illegible]<br>TTAATAGT<br>TAAT	[illegible]<br>GTCCGGGT<br>GTGA	[illegible]<br>GGTGGTAT<br>TGTG	[illegible]<br>TGCTATTA<br>CCTT	[illegible]<br>CTACTGAA<br>ATGC	[illegible]<br>AGCTAAAA<br>GACT	[illegible]<br>GAACTCAA<br>TCAT	[illegible]<br>GCACCATT<br>TTT	[illegible]<br>CTAAAAAG<br>GCTA	[illegible]<br>TTCGCACC<br>ATTT	[illegible]<br>TCAGACTA<br>AGTC	[illegible]<br>CGGTAACA<br>AACC	[illegible]<br>TGTTAACT<br>GCAC	[illegible]<br>TGAACTTG<br>ATG	[illegible]<br>ACAATTTC<br>ACTA	[illegible]<br>CTTTTAAA<br>GTTT	[illegible]<br>ACCCTAAT<br>TATG	[illegible]<br>GTAACTTT<br>AACT
TATGGTGT<br>TGGTCACC<br>AACC	TATGGTTG<br>ATACTAGT<br>TTGA	TATGGTTTC<br>CGACCCAC<br>TTA	TATGTATT<br>GTTCTTTTT<br>ACC	TATGTCAA<br>CAACTCAT<br>ATGA	TATGTCATT<br>CATTGTAC<br>TCT	TATGTGCT<br>AAGCACTA<br>TGTG	TATGTGTT<br>GACGTACC<br>TGGC	TATTAATTT<br>AGGGCGTG<br>ATC	TATTACAG<br>AGGGTAGT<br>GTTA	TATTACCTT<br>TTACGCAA<br>TAT	TATTACTA<br>ATTATTAT<br>GCGG	TATTATGC<br>GGACTTTT<br>AAAG	TATTCCTTA<br>TGTCATTC<br>ATT	TATTCTGTC<br>CTATATAA<br>TTT	TATTGCTG<br>ATTATAAT<br>TATA	TATTGTGG<br>CTATCGTA<br>GTAA	TATTGTTCT<br>TTTTACCCT<br>CC	TATTTACTA<br>ATATGTTT<br>ACA	TATTTATAC<br>AGAACTGG<br>AAC	TATTTTGGT<br>GGTTTTAA<br>TTT	TCAACCAT<br>AATGCACA<br>AGCT
TCAACTCC<br>AGGCAGC<br>AGTAA	TCAAGATG<br>TGGTCAAC<br>CATA	TCAATCAT<br>ACACTAAT<br>TCTT	TCAATGAT<br>GATACTTT<br>CTGA	TCAATGCC<br>AGATTATG<br>TGCT	TCAATTGA<br>GTACAGAC<br>ATTG	TCACCAAC<br>CATACAGA<br>GTAG	TCAGACTA<br>AGTCTCAT<br>CGGC	TCAGACTC<br>AGACTAAG<br>TCTC	TCAGCACA<br>TCTAGGTTT<br>TGT	TCAGCCAA<br>ATTGCTCC<br>AGGG	TCAGCGAA<br>ATGCACTC<br>CGCA	TCAGGCCG<br>GTAACAAA<br>CCTT	TCAGGGTG<br>TTAACTGC<br>ACAG	TCAGTGTG<br>TTAATCTTA<br>TAA	TCATAACT<br>CTCAAAAA<br>GAGA	TCATACAC<br>TAATTCTTT<br>CAC	TCATATGG<br>TTTCCGAC<br>CCAC	TCATCGGC<br>GGGCACGT<br>AGTG	TCATCTAA<br>ACGAACA<br>AACTT	TCATTCATT<br>GTACTCTG<br>TTT	TCATTCGTT<br>TCGGAAGA<br>GAT
TCATTGTA<br>CTCTGTTTA<br>ACA	TCATTTAAT<br>CCAGAAAC<br>TAA	TCATTTGTA<br>ATTAGAGG<br>TAA	TCATTTTTG<br>AACTTGAT<br>GAA	TCCAAAAT<br>CATAACTC<br>TCAA	TCCAAATT<br>TGGTGCAA<br>TTTC	TCCAGGCA<br>GCAGTAAA<br>CGAA	TCCAGGGC<br>AAACTGGA<br>AATA	TCCATGAG<br>CCGTGCTG<br>ACTC	TCCATGTG<br>GTCATTTA<br>ATCC	TCCATTTTT<br>GGATGTTT<br>ATT	TCCCACTT<br>ACAAGTTT<br>TGGA	TCCGACCC<br>ACTTATGG<br>TGTT	TCCGCATT<br>ACGTTTGG<br>TGGA	TCCTATAT<br>AATTTCGC<br>ACCA	TCCTTATGT<br>CATTCATT<br>GTA	TCCTTGAA<br>GAATGGA<br>ACCTA	TCCTTTACG<br>ATCATATG<br>GTT	TCGCACCA<br>TTTTTCGCT<br>TTT	TCGCTTTTA<br>AGTGTTAT<br>GGA	TCGGAAGA<br>GATAGGTA<br>CGTT	TCGTTTCG<br>GAAGAGA<br>TAGGT
TCTAAACG<br>AACAAACT<br>TAAA	TCTAAACG<br>AACATGAA<br>AATT	TCTAACAA<br>GCTTGATT<br>CTAA	TCTACAGT<br>GTTCCCAC<br>TTAC	TCTAGGTTT<br>TGTCCGGG<br>TGT	TCTATCAG<br>GCCGGTAA<br>CAAA	TCTCAAAA<br>AGAGATG<br>GCAAC	TCTCAATG<br>ATGATACT<br>TTCT	TCTCATCG<br>GCGGGCAC<br>GTAG	TCTCTAAA<br>CGAACATG<br>AAAA	TCTCTACA<br>GTGTTCCC<br>ACTT	TCTCTATCA<br>CCTCAGCT<br>GTT	TCTGAAGA<br>TATGCTTA<br>ACCC	TCTGCACC<br>TCTGAAGA<br>TATG	TCTGTCCTA<br>TATAATTTC<br>GC	TCTGTTATT<br>CCTTGTTTT<br>AA	TCTTATAA<br>CCAGAACT<br>CAAT	TCTTATTAC<br>AGAGGGT<br>AGTG	TCTTTATCA<br>GGGTGTTA<br>ACT	TCTTTTTAC<br>CCTCCAGA<br>TGA	TGAAGAAT<br>GGAACCTA<br>GTAA	TGAAGATA<br>TGCTTAAC<br>CCTA
TGAAGCTA<br>AAAGACTG<br>TGTT	TGAAGGTT<br>TATGTGTT<br>GACG	TGAAGTCA<br>GCCAAATT<br>GCTC	TGAATATC<br>ATTTTTGA<br>ACTT	TGAATATG<br>TCAACAAC<br>TCAT	TGAATTAG<br>ATTCATTC<br>AAGG	TGACGTAC<br>CTGGCATA<br>CCTA	TGAGACAC<br>AATTTCAC<br>TATT	TGAGCCGT<br>GCTGACTC<br>AACT	TGAGTACA<br>GACATTGG<br>TGTT	TGATACTA<br>GTTTGAAG<br>CTAA	TGATACTTT<br>CTGACGAT<br>GCT	TGATCCAT<br>TTTTGGAT<br>GTTT	TGATCTGC<br>ACCTCTGA<br>AGAT	TGATGAAG<br>TTTTTAACG<br>CCA	TGATGATA<br>CTTTCTGAC<br>GAT	TGATGGAA<br>CTGGTACT<br>ATTT	TGATGTGC<br>TATTACCTT<br>TTA	TGCAACAA<br>TCCATGAG<br>CCGT	TGCAACCT<br>GAATTAGA<br>TTCA	TGCACCTC<br>TGAAGATA<br>TGCT	TGCACTCC<br>GCATTACG<br>TTTG
TGCAGGTT<br>TTAATTGTT<br>ACT	TGCCAGAT<br>TATGTGCT<br>AAGC	TGCCCTTTT<br>GATGAAGT<br>TTT	TGCGGACT<br>TTTAAAGT<br>TTCC	TGCTATGA<br>GACACAAT<br>TTCA	TGCTATTA<br>CCTTTTACG<br>CAA	TGCTCCAG<br>GGCAAACT<br>GGAA	TGCTGTAA<br>ATTTATTTA<br>CTA	TGCTGTTCT<br>TTATCAGG<br>GTG	TGCTTTTTA<br>CATTCTAC<br>CAT	TGCTTTTTA<br>GCCTTTCTG<br>TT	TGGAAATA<br>TTGCTGATT<br>ATA	TGGAACTG<br>GTACTATTT<br>ATA	TGGAATTC<br>TAACAAGC<br>TTGA	TGGAGATT<br>CTCTAAAC<br>GAAC	TGGATGTT<br>TATTACCA<br>CAAA	TGGCAGTA<br>ACCAGAAT<br>GGTG	TGGCCAGT<br>AACTTTAA<br>CTTG	TGGCCATA<br>GGTACGGC<br>GCCG	TGGCTATC<br>GTAGTAAC<br>ATGC	TGGTACTA<br>TTTATACA<br>GAAC	TGGTATTG<br>TGGCTATC<br>GTAG
TGGTCAAC<br>CATAATGC<br>ACAA	TGGTCACC<br>AACCATAC<br>AGAG	TGGTCATTT<br>AATCCAGA<br>AAC	TGGTGGCC<br>ATAGGTAC<br>GGCG	TGGTGGGG<br>CGCGATCA<br>AAAC	TGGTGGTA<br>TTGTGGCT<br>ATCG	TGGTGTTCT<br>TATTACAG<br>AGG	TGGTGTTG<br>CAGGTTTT<br>AATT	TGGTGTTG<br>GTCACCAA<br>CCAT	TGGTTGAT<br>ACTAGTTT<br>GAAG	TGGTTTCC<br>GACCCACT<br>TATG	TGTAAAAG<br>TGCTTTTTA<br>CAT	TGTAAATT<br>TATTTACTA<br>ATA	TGTAATGA<br>TCCATTTTT<br>GGA	TGTAATGG<br>TGTTGCAG<br>GTTT	TGTAATTA<br>GAGGTAAT<br>GAAG	TGTACACA<br>ATTAAAAC<br>GTGC	TGTAGATG<br>CTGTAAAT<br>TTAT	TGTATTGTT<br>CTTTTTACC<br>CT	TGTCAATG<br>CCAGATTA<br>TGTG	TGTCATTC<br>ATTGTACT<br>CTGT	TGTCCGGG<br>TGTGACCG<br>AAAG
TGTCCTAT<br>ATAATTTC<br>GCAC	TGTGAATA<br>TCATTTTTG<br>AAC	TGTGCCCTT<br>TTGATGAA<br>GTT	TGTGCTAA<br>GCACTATG<br>TGTA	TGTGCTAT<br>GAGACAC<br>AATTT	TGTGCTATT<br>ACCTTTTAC<br>GC	TGTGGCTA<br>TCGTAGTA<br>ACAT	TGTGGTCA<br>ACCATAAT<br>GCAC	TGTGGTCA<br>TTTAATCC<br>AGAA	TGTGTTAA<br>TCTTATAA<br>CCAG	TGTGTTGA<br>CGTACCTG<br>GCAT	TGTTAATCT<br>TATAACCA<br>GAA	TGTTACTTT<br>CCTTTACG<br>ATC	TGTTATTCC<br>TTGTTTTAA<br>TT	TGTTCCCA<br>CTTACAAG<br>TTTT	TGTTCTTAT<br>TACAGAGG<br>GTA	TGTTCTTTA<br>TCAGGGTG<br>TTA	TGTTCTTTT<br>TACCCTCC<br>AGA	TGTTGACG<br>TACCTGGC<br>ATAC	TGTTGCAG<br>GTTTTAATT<br>GTT	TGTTGGTC<br>ACCAACCA<br>TACA	TGTTGTCA<br>ATGCCAGA<br>TTAT
TGTTTAAT<br>AGGGGCTG<br>AATA	TTAAAAAG<br>CTCCTTGA<br>AGAA	TTAAAAAG<br>TGTAAAAG<br>TGCT	TTAAAACG<br>TGCTTTAA<br>CTGG	TTAAACAA<br>CTTAGCTC<br>CAAA	TTAAATAT<br>TTTGGTGG<br>TTTT	TTAAATGT<br>CTGATAAT<br>GGAC	TTAACTTGT<br>TTTGTGCTT<br>GC	TTAATAGG<br>GGCTGAAT<br>ATGT	TTAATCCA<br>GAAACTAA<br>CATT	TTAATCTTA<br>TAACCAGA<br>ACT	TTAATTTA<br>GGGCGTGA<br>TCTC	TTACAAGT<br>TTTGGACC<br>ACTA	TTACAGAG<br>GGTAGTGT<br>TAAA	TTACCAAC<br>CACCACAA<br>ATCT	TTACCAAT<br>GTGCTATG<br>AGAC	TTACCCTCC<br>AGATGAG<br>GATG	TTACCTTTT<br>ACGCAATA<br>TAA	TTACGATC<br>ATATGGTT<br>TCCG	TTACTAATT<br>ATTATGCG<br>GAC	TTACTTTCC<br>TTTACGAT<br>CAT	TTAGAGGT<br>AATGAAGT<br>CAGC
TTAGATTC<br>ATTCAAGG<br>AGGA	TTAGCCTTT<br>CTGTTATTC<br>CT	TTAGCTCC<br>AAATTTGG<br>TGCA	TTAGGGCG<br>TGATCTCC<br>CTCA	TTATAACC<br>AGAACTCA<br>ATCA	TTATACAG<br>AACTGGAA<br>CCAC	TTATCAGA<br>CTCAGACT<br>AAGT	TTATCAGG<br>GTGTTAAC<br>TGCA	TTATGCGG<br>ACTTTTAA<br>AGTT	TTATGGCC<br>AGTAACTT<br>TAAC	TTATGGTG<br>TTGGTCAC<br>CAAC	TTATGTCAT<br>TCATTGTA<br>CTC	TTATGTGCT<br>AAGCACTA<br>TGT	TTATGTGTT<br>GACGTACC<br>TGG	TTATTACA<br>GAGGGTA<br>GTGTT	TTATTATGC<br>GGACTTTT<br>AAA	TTATTCCTT<br>GTTTTAATT<br>AT	TTATTTACT<br>AATATGTT<br>TAC	TTCAAGAT<br>GTGGTCAA<br>CCAT	TTCATTCA<br>AGGAGGA<br>GTTAG	TTCATTGTA<br>CTCTGTTTA<br>AC	TTCATTTGT<br>AATTAGAG<br>GTA
TTCCAAAA<br>TCATAACT<br>CTCA	TTCCATGT<br>GGTCATTT<br>AATC	TTCCCACTT<br>ACAAGTTT<br>TGG	TTCCGACC<br>CACTTATG<br>GTGT	TTCCTTATG<br>TCATTCATT<br>GT	TTCCTTTAC<br>GATCATAT<br>GGT	TTCGCACC<br>ATTTTTCGC<br>TTT	TTCGCTTTT<br>AAGTGTTA<br>TGG	TTCGGAAG<br>AGATAGGT<br>ACGT	TTCGTTTCG<br>GAAGAGA<br>TAGG	TTCTAACA<br>AGCTTGAT<br>TCTA	TTCTCAAT<br>GATGATAC<br>TTTC	TTCTCTAA<br>ACGAACAT<br>GAAA	TTCTCTACA<br>GTGTTCCC<br>ACT	TTCTGACG<br>ATGCTGTT<br>GTGT	TTCTGTCCT<br>ATATAATT<br>TCG	TTCTGTTAT<br>TCCTTGTTT<br>TA	TTCTTATTA<br>CAGAGGGT<br>AGT	TTCTTTATC<br>AGGGTGTT<br>AAC	TTCTTTTTA<br>CCCTCCAG<br>ATG	TTGAAACG<br>ATTTGTGC<br>ACCA	TTGAAGAA<br>TGGAACCT<br>AGTA
TTGAAGCT<br>AAAAGACT<br>GTGT	TTGACGTA<br>CCTGGCAT<br>ACCT	TTGAGTAC<br>AGACATTG<br>GTGT	TTGATACT<br>AGTTTGAA<br>GCTA	TTGATGAA<br>GTTTTTAAC<br>GCC	TTGCAACA<br>ATCCATGA<br>GCCG	TTGCAACC<br>TGAATTAG<br>ATTC	TTGCAGGT<br>TTTAATTGT<br>TAC	TTGCTCCA<br>GGGCAAA<br>CTGGA	TTGCTGTTC<br>TTTATCAG<br>GGT	TTGGAATT<br>CTAACAAG<br>CTTG	TTGGATGT<br>TTATTACC<br>ACAA	TTGGTCAC<br>CAACCATA<br>CAGA	TTGGTGTT<br>GAACATGT<br>TACC	TTGTAATG<br>GTGTTGCA<br>GGTT	TTGTAATT<br>AGAGGTA<br>ATGAA	TTGTACAC<br>AATTAAAA<br>CGTG	TTGTACTCT<br>GTTTAACA<br>CCA	TTGTCAAT<br>GCCAGATT<br>ATGT	TTGTCCGG<br>GTGTGACC<br>GAAA	TTGTGCCCT<br>TTTGATGA<br>AGT	TTGTGGCT<br>ATCGTAGT<br>AACA
TTGTTACTT<br>TCCTTTACG<br>AT	TTGTTCTTT<br>TTACCCTCC<br>AG	TTTAACTTG<br>TTTTGTGCT<br>TG	TTTAATAG<br>GGGCTGAA<br>TATG	TTTAATCC<br>AGAAACTA<br>ACAT	TTTACCAA<br>CCACCACA<br>AATC	TTTACCAA<br>TGTGCTAT<br>GAGA	TTTACCCTC<br>CAGATGAG<br>GAT	TTTACGAT<br>CATATGGT<br>TTCC	TTTACGCA<br>ATATAATA<br>GATA	TTTACTAAT<br>ATGTTTAC<br>ACC	TTTAGCCTT<br>TCTGTTATT<br>CC	TTTAGGGC<br>GTGATCTC<br>CCTC	TTTATACA<br>GAACTGGA<br>ACCA	TTTATCAG<br>GGTGTTAA<br>CTGC	TTTATGTGT<br>TGACGTAC<br>CTG	TTTATTTAC<br>TAATATGT<br>TTA	TTTCCGAC<br>CCACTTAT<br>GGTG	TTTCCTTTA<br>CGATCATA<br>TGG	TTTCGCAC<br>CATTTTTCG<br>CTT	TTTCGCTTT<br>TAAGTGTT<br>ATG	TTTCGGAA<br>GAGATAG<br>GTACG
TTTCTCAAT<br>GATGATAC<br>TTT	TTTCTGAC<br>GATGCTGT<br>TGTG	TTTCTGTTA<br>TTCCTTGTT<br>TT	TTTGAAGC<br>TAAAAGAC<br>TGTG	TTTGATGA<br>AGTTTTTA<br>ACGC	TTTGCAAC<br>CTGAATTA<br>GATT	TTTGGATG<br>TTTATTACC<br>ACA	TTTGTAATT<br>AGAGGTA<br>ATGA	TTTGTACA<br>CAATTAAA<br>ACGT	TTTGTCCG<br>GGTGTGAC<br>CGAA	TTTTACCCT<br>CCAGATGA<br>GGA	TTTTACGC<br>AATATAAT<br>AGAT	TTTTAGCCT<br>TTCTGTTAT<br>TC	TTTTCGCTT<br>TTAAGTGT<br>TAT	TTTTGATG<br>AAGTTTTT<br>AACG	TTTTGGAT<br>GTTTATTAC<br>CAC	TTTTGTACA<br>CAATTAAA<br>ACG	TTTTGTCCG<br>GGTGTGAC<br>CGA	TTTTTACAT<br>TCTACCAT<br>CTA	TTTTTACCC<br>TCCAGATG<br>AGG	TTTTTAGCC<br>TTTCTGTTA<br>TT	TTTTTCGCT<br>TTTAAGTG<br>TTA
TTTTTGAAC<br>TTGATGAA<br>AGG	TTTTTGGAT<br>GTTTATTAC<br>CA	TTTTTGTAC<br>ACAATTAA<br>AAC																			
